# Clinical features, molecular pathology, and immune microenvironmental characteristics of acral melanoma

**DOI:** 10.1186/s12967-022-03532-2

**Published:** 2022-08-16

**Authors:** Jianping Gui, Zhen Guo, Di Wu

**Affiliations:** Cancer Center, The First Hospital of Jilin University, 1 Xinmin St, Changchun, 130021 China

**Keywords:** Acral melanoma, Clinical features, Molecular pathology, Immune microenvironment

## Abstract

Acral melanoma (AM) has unique biology as an aggressive subtype of melanoma. It is a common subtype of melanoma in races with darker skin tones usually diagnosed at a later stage, thereby presenting a worse prognosis compared to cutaneous melanoma. The pathogenesis of acral melanoma differs from cutaneous melanoma, and trauma promotes its development. Compared to cutaneous melanomas, acral melanomas have a significantly lighter mutational burden with more copy number variants. Most acral melanomas are classified as triple wild-type. In contrast to cutaneous melanomas, acral melanomas have a suppressive immune microenvironment. Herein, we reviewed the clinical features, genetic variants, and immune microenvironmental characteristics of limbic melanomas to summarise their unique features.

## Background

Acral melanoma (AM) occurs in the glabrous skin of palms, soles, and nail beds and is the most common melanoma subtype in Asian, African, and Hispanic populations. Notably, due to its unique risk factors, site of origin, and pathological type, AM has significantly different clinical outcomes than cutaneous melanoma (CM). The local recurrence of AM is two to five times higher than other melanoma subtypes [1]. Moreover, AM is usually at a more advanced disease stage when diagnosed. Immune checkpoint inhibitors and targeted therapies have dramatically changed the clinical outcomes of melanoma and have significantly improved the prognosis of melanoma patients. However, AM patients do not benefit as much from targeted therapy and immunotherapy as CM patients. Hence, since melanoma treatments enter the era of targeted and immunotherapy, it is particularly important to investigate the molecular and immunological features of AM pathogenesis.

## Clinical characteristics

The causative factors of AM are different from CM, and AM is less likely to develop from ultraviolet damage, due to the low exposure to sunlight on the palms, soles, and nail beds. Moreover, the nail plate has been shown to protect the skin against ultraviolet exposure but this protection might be incomplete [2]. Trauma is a controversial potential cause of extremity melanomas and some studies have not found statistically significant differences in trauma groups [3]. However, AM tends to occur in the foot, suggesting the possibility that trauma and mechanical stress contribute to its development [1, 4, 5]. Additionally, a retrospective study of a Chinese population found a potential association between trauma and AM, particularly lower limb melanoma [6]. The 104 cases of trauma-related melanoma had a significant predominance of AM and the risk of post-trauma melanoma was significantly higher in the upper and lower extremities than in other sites (*p* < 0.0001) [6]. A Korean study analyzing the relationship between AM and trauma reached similar conclusions [7]. Nevertheless, the relationship between trauma and AM remains unclear. Trauma does not necessarily lead to AM development, but its effects on AM cannot be ignored. Although there is no clear statistical evidence, the relationship between trauma and the development of nail apparatus melanoma (NAM) is agreed upon by most researchers [8–10]. Some studies have even suggested that trauma has a much greater impact on NAM than AM since NAM occurs more often in the thumb or big toe (75–90%), which are more susceptible to trauma [11]. One study has also found that NAM is more closely associated with trauma than non-nail acral melanoma (NNAM) (*p* = 0.002) and that nails are most often affected by trauma, followed by toenails [7].

Interestingly, there are differences in the incidence of AM between men and women across ethnic groups. However, some studies did not find a significant difference regarding gender in AM [12–14] and there are conflicting views on the relationship between gender and AM prognosis since studies have found that gender is not an independent prognostic factor of AM [15–17]. In contrast, other studies have suggested that being a woman is an independent prognostic factor, presenting prolonged overall survival (OS) compared to male patients. Being a male is also associated with a poorer prognosis [16], and a study analyzing Caucasian versus Chinese AM patients found that the 5-year disease-specific survival (DSS) was 77.9% in Chinese female patients compared to 59.4% in male patients, after controlling for other influencing factors. Similar results were observed in the Caucasian group [18]. These results might be due to the thicker Breslow thickness in males at the time of diagnosis [19]. In another study with ALM patients, the 5- and 10-year DSS rates were higher in women than in men (*p* < 0.001), and men were more likely to develop thicker tumors than women (*p* < 0.001). Moreover, men had later disease staging than women (*p* < 0.001) [19], as described by Phan, Kolla, and Huang et al. [4, 20, 21]. Additionally, in a study of melanomas located in the lower limbs, the frequency of disease progression was higher in men than in women, regardless of whether the site of disease was in the legs or feet. Besides, in the foot group, lymph node involvement was more frequent in men. The authors hypothesized that this difference might be related to the different lymphatic drainage of the foot in men and women [22].In a retrospective study with 176 ALM patients, 60.30% of patients who underwent sentinel lymph node (SLN) biopsy were positive and negative SLN patients were predominantly females (1:4) [15]. The relationship between gender and the clinical outcome of AM remains a matter of debate and research, and hormonal or immunological factors might be responsible for these differences.

Furthermore, AM has a variable incidence across ethnic groups. It is the most common subtype of melanoma in Asian, African, and Hispanic populations. The most common melanoma subtypes in China are AM and mucosal melanoma, accounting for approximately 65% of cases [23]. Moreover, NAM, an AM subtype, accounts for 0.7–3.5% of melanomas in Caucasians [24], with a higher incidence (10–75%) in Asian and African patients [10, 25]. The clinical outcomes of AM also vary among races. A study with 4139 acral melanoma patients found that black, Asian, and Hispanic patients exhibited more advanced disease staging, had thicker Breslow thickness, and had more ulcers compared to Caucasians. This study also found that income, education, and social welfare were statistically significant to prognosis in the black and Hispanic populations. These factors might also contribute to delayed diagnosis in patients, which is related to worse OS [26].

Additionally, Huang et al. have shown that Chinese patients have more advanced diseases compared to Caucasians, including thicker Breslow thickness and more ulcers. However, after controlling for staging and Breslow thickness, the 5-year DSS rates were 68.4 and 73% for Chinese and Caucasian AM patients, respectively, with no significant difference (*p* = 0.56) [18], similar to Bradford et al. Additionally, there were no statistically significant differences in the 5- and 10-year survival rates between races (non-Hispanic whites, blacks, Hispanic whites, and Asian/Pacific Islanders) after controlling for tumor thickness or ALM stage (*p* > 0.05) [27]. These studies have found differences in disease stage, thickness, and ulcer rates between races, but not in the stratified analysis. Possibly, the genetic alterations in AM patients are similar between races, and the differences in prognosis might be associated with delay in diagnosis. Nevertheless, other socioeconomic factors might also influence prognosis.

Non-nail acral melanomas are more common on the foot (82–88.6%) [4, 5, 15] and nail melanoma appears to be more common on the nails than on the toenails (58–61%) [7, 12, 15, 20]. The prognosis for AM is worse than for CM. Currently, there is debate in the literature as to whether this poorer prognosis is due to the more aggressive biology of AM, its unique site of origin, or the late clinical stage at diagnosis. Many researchers consider the foot as an independent risk factor for clinical outcomes (Table 1). For example, in melanomas with lower limb sites, the prognosis is significantly higher in the leg group than in the foot group, regardless of the histological subtype [22, 28]. Additionally, a study found that plantar melanoma had a worse prognosis compared to the palms and nail beds [29]. Thus, poorer AM prognoses might be more closely related to the anatomical site than the histological subtype [5], similar to NAM. For example, Kostaki et al. found that tumors in the toes had a higher Breslow thickness at diagnosis compared to those in the fingers (*p* < 0.001) [30]. The authors hypothesized that since lesions in the hand are more easily detected finger melanoma patients are diagnosed earlier. Additionally, a retrospective study with NAM patients in Brazil found that melanomas occurring in the toes had worse 5-year relapse-free survival (RFS) compared to finger melanomas, with the anatomical location of the foot being an independent risk factor [31].

**Table 1:** Influence of the location on the prognosis of acral melanoma

First author	Study type	Cases	Location	Results	Ref.
Gavillero A	Retrospective study	n = 285 (SSM and NM on the lower limb)	Foot vs. Leg	Multifactorial and univariate analyses confirmed the foot location as an independent prognostic factor associated with reduced melanoma-specific survival (HR of 2.3 and 2.7, respectively)	[28]
Martina Sanlorenzo	Retrospective study	n = 1671 (Total); n = 327 (Foot)	Foot vs. Leg	Multifactorial and univariate analyses confirmed the foot site as a negative independent prognostic factor for disease-specific survival (HR of 2.53 and 1.52 respectively)	[22]
Xiaoting Wei	Multi-center retrospective study	n = 1157; n = 792 (Soles); n = 95 (Palms); n = 270 (Nail beds)	Sole vs. Nail bed vs. Palm	The 10-year survival rates were 32.8%, 60.4%. and 48.9% for sole, palm, and nail bed groups, respectively. The median MSS of patients in the sole group was only 65.0 m, significantly shorter than in the nail bed (112.0 m) and palm group (NR) (*p* = 0.0053)	[29]

*SSM* superficial spreading melanoma, *NM* nodular melanomas, *NR* not reach

## Liquid biopsy

In recent years, liquid biopsy has been widely used in melanoma. Related studies have found that the number of CMCs (circulating melanoma cells) correlates with the occurrence, and invasion of melanoma. The number of CMCs is significantly higher in patients with metastatic melanoma [32]. The levels of the melanoma cell adhesion molecule MCAM (MUC18/MelCAM/CD146) are also correlated with tumor aggressiveness [33]. The 12-month PFS rates are significantly better in melanoma patients with PD-L1-positive CTCs(circulating tumour cells) than in negative patients (81% vs 22%) [34].

Increased ctDNA concentrations are also associated with poorer OS [35] and ctDNA (circulating tumour DNA) assays can be used to assess the response of melanoma patients to drug therapy. The levels of ctDNA [BRAF (V600E), BRAF (V600K), or NRAS (Q61H)] decrease when there is a response to targeted therapy and increase as the disease progresses [36]. Moreover, when melanoma patients become resistant to BRAF/MEK inhibitors, increased copy numbers of MET mutations is detected in the ctDNA [37].

There is also a link between exosomes and drug resistance in melanoma [38]. Exosomes can be involved in the growth and survival of cancer cells through propagated resistance. PDGFRβ moves to melanoma cells via exosomal transport and activates the phosphatidylinositol-3-kinase (PI3K-AKT) signaling pathway, thereby reducing the susceptibility to BRAF inhibitors [39]. The levels of PD-L1 on melanoma-derived exosomes are associated with poor disease prognosis, Exosomes carrying PD-L1 had immunosuppressive properties, and that can mediate tumor-induced immunosuppression [40].

Finally, circulating miRNAs can be used as biomarkers for melanoma diagnosis. For example, the deletion of miR-29c and miR-324-3p in the serum of melanoma patients suggests an association with melanoma metastasis [41]. Additionally, the upregulation of miR-221 and miR-10b expression is associated with poor prognosis [42, 43].

Overall, these studies have demonstrated the importance of liquid biopsy as a tool for melanoma diagnosis, efficacy prediction, and prognosis determination.

## Mutational landscape

Compared to CM, AM has more chromosomal structural variations and copy number variations (CNVs) [23, 44]. Tumour mutation burden (TMB) in cutaneous melanoma is more than 18 times in acral melanoma {Hayward, 2017 #321}. The accumulation of chromosomal instability occurs at the initial stage of AM, followed by KIT, BRAF, and NRAS mutations and other rare driver mutations [44, 45]. BRAF (10–35%) and NRAS (8–27.9%) mutations are common driver mutations in AM but are much less frequent than in CM [BRAF (45–50%) and NRAS (19–30%)]. Besides, the proportion of triple wild-type (TWT) mutations that do not express BRAF, NRAS, or NF1 mutations is higher in AM than in CM (38%vs11%) [46]. Moreover, NF1 and KIT (6%-20.7%) [47, 48] mutations and amplification of CCND1, CDK4, MITF, and TERT are also common in AM [49–51]. In NAM, BRAF and NRAS frequencies are low [51–53], and KIT mutations are more common [44]. Holman et al. found that KIT mutations are more common in NAM (16%) than in NNAM (3%), with BRAF and NRAS mutations occurring almost exclusively in NNAM [54]. This result was also supported by Elefanti et al. They also found that TWT was closely associated with NAM [55]. Additionally, one study found significant amplification of a region in chromosome 4, including KIT, in NAM patients, whereas no such mutations were observed in NNAM [46].

Furthermore, BRAF and NRAS mutations might be associated with ultraviolet radiation (UVR)-induced damage [56], which would partly explain the lower BRAF and NRAS mutation rates in AM compared to CM. Previous studies have proposed a classification of AM based on the BRAF V600E mutation. BRAF V600E-mutant AMs are similar to low Chronic sun damage (low-CSD) melanomas, presenting fewer DNA copy number changes, whereas the histological subtype of non-BRAF V600E-mutant patients is more likely to be ALM [51]. Newell et al. also found that BRAF V600E-mutant AMs are similar to CMs with low rearrangement burden and fewer samples with complex chromosomes [46]. Additionally, BRAF-mutated AM has been associated with earlier clinical staging (pT1-T2 stages), more favorable histological prognostic factors (such as thinner Breslow thickness), and lower mitotic counts [55]. The clinical outcomes of BRAF-mutated AM patients are also better than in wild-type BRAF patients [57]. The BRAF mutations are also common in benign nevi [58]. However, Yamazaki et al. showed that BRAF V600 mutations are more common in advanced ALM than in early ALM [59]. Moreover, BRAF mutations participate in the metastatic spread of melanoma [60]. Overall, these studies have indicated that BRAF mutations play an important role in melanoma development, maintenance, and progression.

Previous studies have found that AM with UVR characteristics most often occurs in the nail area [2], suggesting that the nail plate is not completely resistant to UVR. A study with 87 tumor tissue specimens (59 tumors from the soles of the feet, 6 from the palms of the hands, and 22 nail tumors) found that nail tumors had a higher proportion of UVR features than toenail tumors. Besides, NAM presented the highest mutational burden of all tissue specimens, while foot NNAM presented the lowest [46]. Finally, Shi et al. showed that foot NAM has a higher mutational load than foot NNAM [44].

Acral melanoma has complex and variable chromosomal structural abnormalities, including copy number amplification and deletion, chromosomal aneuploidy, and localized structural rearrangements. The gain of chromosomes 7 and 8 and loss of chromosomes 9 and 10 has been previously identified by Newell et al. Additionally, isochromosomes consisting of 6p gain and 6q loss are more common in NAM [46]. The investigators also observed recurrent complex rearrangements on chromosomes 5, 6, 7, 11, and 12, associated with amplification of TERT, CDK4, MDM2, CCND1, PAK1, and GAB2 [46]. Yeh et al. found that PAK1 and GAB2 on the long arm of chromosome 11 were within 1 Mb of each other and were always co-amplified. MDM2 on chromosome 12 was co-amplified with CDK4 in more than a third of CDK4 amplification cases, and EP300 was amplified on chromosome 22 [51]. Numerous genome sequencing results on AM have identified common copy number amplified genes, including CCND1, GAB2, PAK1, TERT, YAP1, MDM2, CDK4, NOTCH2, KIT, and EP300; and copy number deletion regions, including those containing CDKN2A and NF1, and PTEN [47, 61].

Moreover, TERT amplification can be associated with poor AM prognosis. For example, Yu et al. determined TERT amplification as an independent poor prognostic factor for RFS in AM patients treated with high dose interferon (HD-IFN) [62].

The frequency of EP300 gains is also higher in melanomas than in CM (24.5% vs. 11.75%) [44]. Shi et al. have shown that, in patients carrying increased copy numbers of the EP300-MITF axis, AM is more aggressive than in patients without these variants, besides presenting thicker Breslow thickness, more ulcers, and later clinical staging. Furthermore, EP300 gains are associated with a suppressive inflammatory environment, as evidenced by reduced expression of pro-inflammatory genes (IL8, IL1B, IL1RN, and Ptgs2) [44]. This might be associated with immune escape from AM. For example, a study has previously determined mutations associated with AM invasion and metastasis, including EP300, ANO1, CPEB1, INADL, MAP1B, MAP7D1, MARCH 6, NETO1, PRKCE, SBK1, TNRC6A, USP13, WDR74, and ZNF827 [63]. Farshidfar et al. have found that recurrent, late-arising focal amplifications of cytoband 22q11.21 associated with limbic melanoma metastasis was a major determinant of poor clinical outcome and was related to the downregulation of immunomodulatory genes associated with immunotherapeutic response. For example, LZTR1 and CRKL are two important genes associated with 22q11.21 amplification in limbic melanoma, and LZTR1 can be a viable therapeutic target [64].

In previous studies, NAM presented the most diverse group of oncogenic mutations, including KRAS, CTNNB1, TP53, ERBB2, SMAD4, PIK3CA, STK11, EGFR, FGFR3, and PTPN11 mutations [52, 53]. The genome of NAM has significantly more CNVs than NNAM [52, 65]. Lim et al. suggested that mutations in CSMD3 and EHMT1 might play a significant oncogenic role in NAM, but not in NNAM [66]. Holman et al. have found that, in the PI3K/mTOR pathway, RICTOR and TSC1 alterations are prevalent in NAM, while AKT1 alterations and PTEN loss are common in NNAM [54]. Additionally, NAM and NNAM have their preferred pathogenic pathways, such as DNA replication and repair pathways as well as chromatin modification pathways, although not statistically significant [54].

Amplification of CCND1 and loss of CDKN2A can activate the CDK4 pathway, which is a common genetic feature of AM [51]. Kong et al. found genetic aberrations of the CDK4/6 pathway in 82.7% of AM cases. They further showed that patients with CDK4 pathway aberrations had a significantly worse prognosis compared to those without these aberrations. This might be partly because AM patients with CDK4 pathway aberrations have thicker Breslow thickness and more ulcers [67]. The genetic variants in the CDK4 pathway have also been associated with innate resistance to PD-1 therapy in non-CM patients [68], providing some theoretical support for the poorer response of AM to immunotherapy.

In melanomas lacking these common coding mutations (BRAF, NRAS, KRAS, HRAS, NF1, KIT, GNAQ, and GNA11), there is a high frequency of kinase fusions [69]. These fusions might play a specific role in tumor development [70]. In AM, kinase fusions include PAK1, DGKB, NTRK1, BRAF, ALK, and RET [49, 69], and might be potential therapeutic targets.

## Tumor immune microenvironment (TIME)

Tumor-infiltrating lymphocytes (TILs) are a fundamental component of the TIME, representing a population of lymphocytes with specific immune responsiveness to tumor cells [71]. Many studies have explored the role of TILs as biomarkers of tumor immune responses in melanoma [72–74]. For example, it has been suggested that TILs, particularly cytotoxic CD8 + T cells, are a prognostic factor for the OS in melanoma patients and are also associated with the response to immune checkpoint inhibitors (ICIs) [71]. For example, a study with AM patients demonstrated that high TILs can be associated with a good survival prognosis [75], similar to Castaneda et al. [76, 77]. Moreover, AM has a lower response rate to ICIs compared to CM [78, 79]. A small Japanese clinical study also found that NAM responded worse to immunotherapy than NNAM [79]. AM also has a suppressive immune microenvironment compared to CM [80] (Table 2). Another study compared the differences in the levels of infiltrating lymphocytes and programmed death receptor-1 (PD-1) in various melanoma subtypes and found lower levels of TILs and PD-1 in NAM tumors than in NNAM [81], which might partly explain the poorer response of NAM to immunotherapy. However, the sample size of this study was small and more research is needed. Nuclear factor κB (NF-κB), a protein complex associated with tumor cell proliferation, invasion, and anti-apoptosis, has an invasive role in AM by reducing the number of CD8+ T cells. Additionally, positive immune expression of NF-κB might be a predictor of increased risk of AM metastasis [82]. Moreover, CD103+ T lymphocytes are significantly associated with infiltration thickness in AM (*p* = 0.0001). However, the immunoexpression of E-calcineurin, a ligand for CD103 and a marker of tumor progression, is not significantly associated with the infiltration thickness of AM [83].

**Table 2:** A *k*ey summary of the immune microenvironment in acral melanoma

First author	Study type	Case	Methodologies	Results	Ref.
Yoshiyuki Nakamura	Retrospective study	CM (n = 53) AM (n = 65)	Immunohistochemistry	The total TIL count was significantly lower in ALM than in CM (54.2 vs. 72.9, *p* < 0.01) The CD8 TIL count was significantly lower in ALM than in CM (33.0 vs. 46.5, *p* < 0.01)	[84]
Jiannong Li	Retrospective study	AM (n = 8); GSE115978 (n = 32); GSE72056 (n = 19) TCGA: AM(n = 336); CM(n = 443)	scRNA-seq	Compared to the non-AM dataset (GSE115978 and GSE72056), acral melanomas had significantly fewer PDCs, CD8 T cells, and NK cells, very few γδ T cells, and a lower mean immune infiltration rate (39.1% vs. 71.2% vs. 67.6) Further validation of the TCGA dataset revealed that the proportion of CD8T effector memory cells, NK cells, and γδ T cells were lower in AM patients than in those with CM	[80]
Miguel Zúñiga-Castillo	Retrospective study	ALM (n = 67); SSM (n = 67)	Immunohistochemistry	Increased M2-Ms in ALM compared to SSM	[85]

CM: cutaneous melanoma; AM: acral melanoma; ALM: acral lentiginous melanoma; SSM: superficial spreading melanoma; GSE115978, GSE72056: the two datasets mentioned in the author’s article.

Furthermore, M2-macrophages (M2-Ms) in the TIME can be associated with local progression, aggressiveness, metastasis, and poor prognosis of melanoma. CD163 is considered a specific marker for M2-Ms [86]. The Zúñiga-Castillo team has demonstrated for the first time that the density of M2-Ms is higher in the tumor microenvironment of ALM compared to superficial spreading melanoma (SSM) [85]. In this study, the density of M2-Ms was positively correlated with Breslow thickness, ulceration, and mitotic activity of ALM patients [85]. These results provided a rationale for the more aggressive biological behavior of AM compared to CM.

Furthermore, CM has high levels of tumor mutational burden (TMB). Many researchers believe that the different responses to immunotherapy between AM and CM might be related to their significant difference in TMB. The higher the TMB of a tumor, the higher the level of neoantigen produced by the tumor and the stronger the T-cell and anti-tumor responses when recognized by the immune system [71]. Thus, tumors with high TMB are usually more immunogenic than those with low TMB [81, 87]. However, data regarding the relationship between TMB and immune infiltration in the TIME are often contradictory, with higher mutation rates not necessarily equating to higher immune infiltration, and with limitations to predict the efficacy of immunotherapy. Cancers with microsatellite instability (MSI) or mismatch repair (MMR) deficiency had high response rates to ICIs. Although a high TMB is common in melanoma, high microsatellite instability (MSI- high, MSI-H) is rare. For example, no significant elevations of MSI levels have been detected in AM samples by Shi et al., and MMR might not be associated with AM in Asian populations [44].

PD-L1 has been suggested as a biomarker to predict the prognosis of melanoma patients [88, 89]. For example, Yun et al. have found that the combined analysis of PD-L1 and TILs can be used to predict the survival outcome of melanoma patients [90]. However, Ren et al. have shown that the levels of PD-L1 in TILs might have a different prognostic value than its levels in tumor cells. However, the levels of PD-L1 in TILs are a poor prognostic factor for primary AM patients [91]. In contrast, another study found that the relationship between the levels of PD-L1 in TILs and survival was not statistically significant, which the authors believed to be related to the different subtypes of melanoma included in the study and differences in PD-L1 assays [90]. Moreover, the levels of PD-L1 can vary among melanoma subtypes. For example, Kaunitz et al. found that the levels of PD-L1 were observed in 31% of AM and 62% of Chronic sun damage patients [92].

## Conclusions

Acral melanoma is a more malignant subtype of melanoma. It is less likely to develop at sites affected by UV damage, and trauma might participate in its development. Gender (males) and the location of the origin site (foot) can be associated with poorer prognoses. Differences in prognosis between races are more likely related to culture, social welfare, and other ethnic backgrounds. Therefore, among people of color, greater emphasis should be placed on melanoma screening and increased protection awareness, which is essential to improve survival outcomes.

**Fig. 1:**
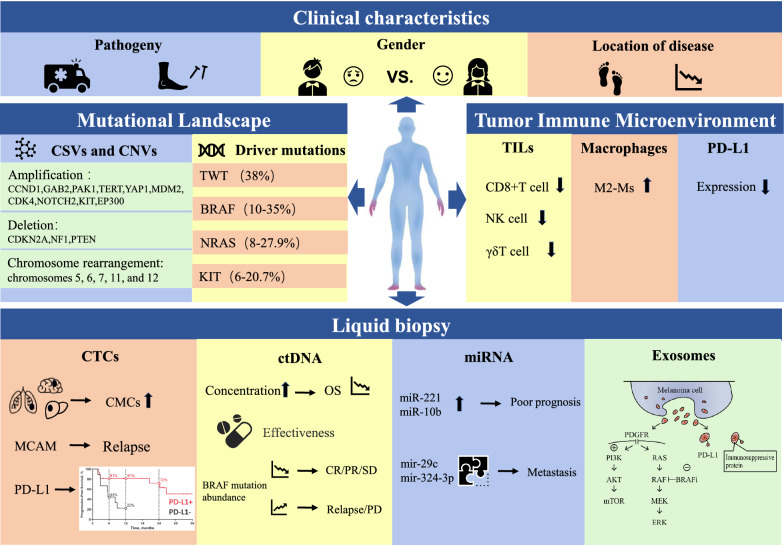
A summary of the main points in the text. *CSVs* chromosomal structural variations, *CNVs* copy number variations, *TWT* triple wild-type, *TILs* tumor infiltrating lymphocytes, *CTCs* circulating tumor cells, *CMCs* circulating melanoma cells, *MCAM* melanoma cell adhesion molecule, *CR* complete response, *PR* partial remission, *SD* stable disease, *PD* progression disease

Whole-genome sequencing studies with AM have revealed a unique genomic profile characterized by variable chromosomal structural variations and low mutational load. The mutation types of AM are more likely to be triple wild-type and, although treatment responses in AM do not significantly differ from CM, targeted therapies are less suitable for AM. Melanoma is considered one of the most immunogenic tumors, and several studies have indicated that the TIME is more suppressive in AM than in CM. Moreover, the response of AM patients to immunotherapy is lower compared to CM patients (Table 3 and Fig. 1). Finally, immune combination therapies are more likely to provide long-term clinical benefits for AM patients.

**Table 3:** A summary of the main clinical features, molecular pathology, and immune microenvironmental characteristics of acral melanoma

Characteristics	Classification	Results/Conclusions	Ref.
Clinical characteristics	Etiology	Trauma may promote the development of extremity melanoma	[1, 6, 7]
	Gender	Men may have a worse prognosis compared to women	[4, 27, 16, 93, 21, 20]
	Anatomic subsite	The poorer prognosis of AM might be more closely related to the anatomical site than the histological subtype	[5, 22, 29, 28, 31]
Molecular pathology characteristics	Chromosomal structural variations and copy number variations	Compared to CM, AM has more chromosomal structural variations and CNVs Common copy number amplified genes include CCND1, GAB2, PAK1, TERT, YAP1, MDM2, CDK4, NOTCH2, KIT, and EP300; common copy number deletion regions, including those containing CDKN2A and NF1 and PTEN	[23, 44, 46, 51, 47, 61]
	Driver mutations	the proportion of TWT mutations is higher in AM than in CM (38% vs. 11%)	[60]
Immune microenvironmental characteristics	TILs	AM has a suppressive immune microenvironment compared to CM (CD 8 + T cell, NK cells, and γδ T cells)	[80, 84]
	M2-Ms	the density of M2-Ms is higher in the ALM tumor microenvironment compared to SSM	[85]
	PD-L1	Lower levels of PD-L1 are present in AM than in chronic sun-damaged melanoma (31% vs. 62%)	[92]

*CM* cutaneous melanoma, *AM* acral melanoma, *CNVs* copy number variations, *TWT* triple wild-type, *Ms* macrophages, *SSM* superficial spreading melanoma

## Data Availability

Not applicable.

## Abbreviations

AM: Acral melanoma
CM: Cutaneous melanoma
ALM: Acral lentiginous melanoma
NAM: Nail apparatus melanoma
NNAM: Non-nail acral melanoma
CMCs: Circulating melanoma cells
CTCs: Circulating tumour cells
CtDNA: Circulating tumour DNA
OS: Overall survival
DSS: Disease-specific survival
SLN: Sentinel lymph node
DFI: Disease-free interval
RFS: Relapse-free survival
CNVs: Copy number variations
TMB: Tumor mutational burden
TWT: Triple wild-type
HD-IFN: High dose interferon
TIME: Tumor Immune Microenvironment
TILs: Tumor-infiltrating lymphocytes
ICIs: Immune checkpoint inhibitors
PD-1: Programmed death receptor-1
NF-κB: Nuclear factor κB
M2-Ms: M2-macrophage
SSM: Superficial spreading melanoma
MSI: Microsatellite instability
MMR: Mismatch repair
UVR: Ultraviolet radiation
CSD: Chronic sun damage
CSVs: Chromosomal structural variations
MCAM: Melanoma cell adhesion molecule
CR: Complete response
PR: Partial remission
SD: Stable disease
PD: Progression disease
